# The First Records of the In Silico Antiviral and Antibacterial Actions of Molecules Detected in Extracts of Algerian Fir (*Abies numidica* De Lannoy) Using LC-MS/MS Analysis

**DOI:** 10.3390/plants13091246

**Published:** 2024-04-30

**Authors:** Djamila Benouchenne, Ines Bellil, Samira Bendjedid, Ana Ramos, Gema Nieto, Salah Akkal, Douadi Khelifi

**Affiliations:** 1 Laboratoire de Génétique Biochimie et Biotechnologies Végétales, Faculté des Sciences de la Nature et de la Vie, Université Frères Mentouri Constantine 1, Constantine 25000, Algeria; d.benouchenne@ensbiotech.edu.dz (D.B.); dkhelifi@yahoo.fr (I.B.); bines07@yahoo.fr (D.K.); 2 Higher National School of Biotechnology, Taoufik KHAZNADAR, Nouveau Pôle Universitaire Ali Mendjili, BP. E66, Constantine 25100, Algeria; 3 Research Laboratory of Functional and Evolutionary Ecology, Department of Biology, Faculty of Natural Sciences and Life, Chadli Bendjedid University, El Tarf 36000, Algeria; samiraphyto@gmail.com; 4 Neuroscience and Aging Research Group, Faculty of Health Sciences, Corporación Universitaria Remington, Calle 51 n°51-27, Medellin 050012, Colombia; 5 Department of Food Technology, Food Science and Nutrition, Faculty of Veterinary Sciences, Regional Campus of International Excellence “Campus Mare Nostrum”, University of Murcia, Espinardo, 30071 Murcia, Spain; 6 Laboratory of Phytochemistry, Natural Products and Organic Synthesis (Physynor), Department of Chemistry, Faculty of Exact Sciences, University Brother Mentouri Constantine 1, Constantine 25000, Algeria; salah4dz@yahoo.fr

**Keywords:** Algerian fir needles, ethyl acetate extract, *n*-butanol extract, molecular docking, antiviral, antibacterial

## Abstract

(1) Background: Due to the wide application in medicinal and pharmaceutical chemistry of flavonoid molecules, which are one of the most famous types of secondary plant metabolites, our work has come within the framework of bio-consulting to help in the identification of the molecule(s) responsible for the antibacterial effect which will be the active principle of a natural antibiotic developed from Algerian fir using bioinformatics tools. (2) Methods: The docking method was used to test the antiviral activity on SARS-CoV-2 virus and the antibacterial activity on Gram-positive *Staphylococcus aureus* and Gram-negative *Escherichia coli* of 12 polyphenolic molecules present in the ethyl acetate and *n*-butanol extracts of Numidian fir leaves, and identify the molecules responsible for these specific biological activities. (3) Results: The findings revealed that it is possible that two molecules, hyperoside and quercitrin, have a high capacity to inhibit SARS-CoV-2, and it is important to mention that they are the most quantitatively abundant molecules in the extract. The molecule luteolin-7-glucoside is probably responsible for the antibacterial activity in the extract against Gram-negative bacteria such as *Escherichia coli*, and the molecule hesperidin is responsible for the antibacterial activity in the extract against Gram-positive bacteria such as *Staphylococcus aureus*.

## 1. Introduction

Phytochemicals, also known as secondary metabolites, are a rich and varied class of aromatic, non-nutritive compounds found in the kingdom of plants. They play a crucial part in the defense mechanism that plants use to fend off biotic factors like pathogens and abiotic stresses like UV radiation [1]. The spread of various bacterial infection-related diseases is a major worry for humanity due to the resistance of microorganisms to antibiotics. The food industry is very interested in using plants’ bioactive molecules to develop and produce functional products, with the goal of eliminating or reducing risks. Because of this, there is growing interest in looking for and finding new products made of synthetic drugs and natural resources.

Due to its geographic location, Algeria has a high biodiversity of plants; the majority of these are endemic and little is known about them. One such example is the Algerian fir (*Abies numidica* de Lannoy ex Carrière) [2], a conifer that is native to Algeria and a member of the Pinaceae family. Its original range is 2300 hectares, and it is found in the Babor Mountains, which are located north of Setif, Algeria. The tree is extremely branchy, with needles wrapped around the branches [3]. It has been used as an anti-inflammatory, to treat respiratory system issues, and as a cataplasm in traditional medicine [4].

To the best of our knowledge, not much is known about this plant and not many papers have been published on it. According to Tlili-Ait Kaki et al.’s [4] GC-MS results, the essential oil extracted from Algerian fir needles collected in Annaba, Algeria, contained high concentrations of bornyl acetate, camphene, alpha-pinene, and beta-pinene. In 2016, Ramdani et al. [5] reported the antimicrobial qualities of essential oil extracted from needles taken from the Babor Mountains in Setif, Algeria. Ghadbane et al. [6] discussed the chemical composition and antimicrobial activity of fractions extracted from *A. numidica* de Lannoy leaves collected from the Babor Mountains. Belhadj Mostefa et al. [7] reported specific diterpenes from *A. numidica* de Lannoy ex Carrière cones in 2017. The potential α-glucosidase inhibitory effect of essential oil extracted from *A. numidica* de Lannoy ex Carrière was reported by Benouchenne et al. [8]. Benouchenne et al. [9] demonstrated that the ethyl acetate fraction derived from the leaves of *A. numidica* exhibited potent antioxidant properties. Benouchenne et al. in 2022 [10] reported on the GC-MS chemical profile and biological activities of essential oil extracted from *Abies numidica* needles. Furthermore, Benouchenne and her team [11] disclosed the tyrosinase inhibitory ability and the in vitro, in vivo, and in silico toxicity of Algerian fir leaf extracts.

A wide range of innovative technologies and techniques have been applied to the use of medicinal plants in recent years, driven by an increasing understanding of the structure and function of compounds [12]. It would be beneficial to be able to predict a large number of chemical compounds accurately in a timely and practical manner [13]. It would take a lot of work and time to evaluate the activities of these ingredients using traditional methods [14].

Determining and forecasting the pharmacological underpinnings of medicinal plant action is crucial to modernizing their application. Because of their complex and varied chemical constituents, it is difficult to identify the precise chemical components and major biological roles of medicinal plants. In traditional medicinal plant research, the extraction of compounds or fractions is typically the first step, followed by their qualitative and quantitative identification [15,16]. The entire research process is generally costly and time-consuming [17]. There are ways to improve efficiency by adjusting different aspects of this shared method. The in-silico method of determining the combinations of simulated compounds and targets has become more accurate as computer technology continues to advance [18]. Thanks to the development of network pharmacology technologies, complex relationships between compounds and their various activity targets can be discovered quickly.

This study aimed to predict, for the first time via an in-silico method, the antiviral and antibacterial effect of molecules detected in ethyl acetate and *n*-butanol fractions of Algerian *Abies numidica* De Lannoy using LC-MS/MS analysis.

## 2. Results

### 2.1. Molecular Docking

#### 2.1.1. Antiviral Ability: Anti-SARS-CoV-2

**Figure 1:**
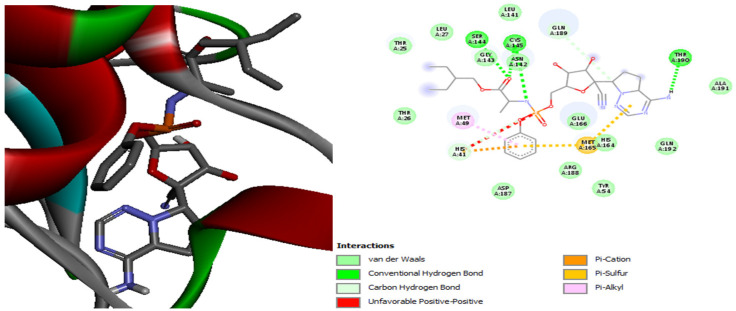
The interaction of the Remdesivir standard with the residue of the active site of the Mpro protein of COVID-19 virus.

Figure 1 shows the interaction of the Remdesivir standard used with the residue of the active site of the Mpro protein. The results revealed that the active site residues of the Mpro protein interacted with the Remdesivir ligand in seven different positions, which were Ser144, Thr190, Met165, Met49, His41, Cys145, and Gln189.

In order to identify novel and more effective COVID-19 Mpro protein inhibitors, we docked 12 polyphenol molecules with the SARS-CoV-2 receptor in this section of the research. The COVID-19 docking server was used (Table S1).

**Figure 2:**
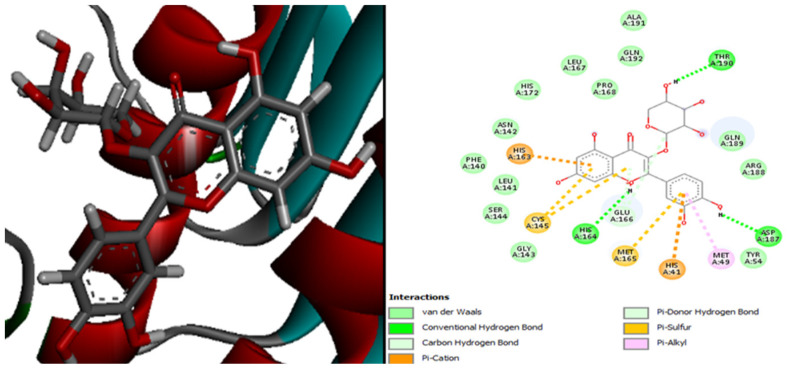
The quercitrin molecule’s superposition and intermolecular interactions in the complex with the COVID-19 virus’s main protease (Mpro).

**Figure 3:**
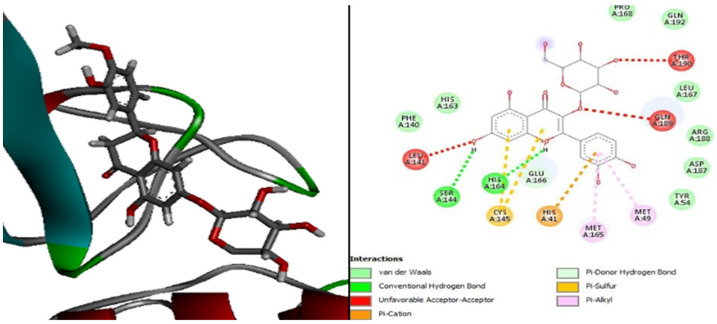
The positioning of the hyperoside molecule and intermolecular interactions within the complex with the primary COVID-19 virus protease (Mpro).

Thr190, Asp187, Met165, Met49, His41, His164, Cys145, Cys145, and His163 are the Mpro residues involved in the nine linkages of the residues that demonstrate the Mpro-Quercitrin complex’s increased stability, as shown by the results displayed in Figure 2. Thr190, His41, Met49, and Met165 are common interactions between the Mpro-Quercitrin complex and Mpro-Remdesivir. However, the Mpro-Hyperoside complex, which includes the following Mpro residues: Thr190, Gln189, Met165, Met49, His41, His164, Cys145, Leu141, and Ser144, was 10 bonds stronger (Figure 3). Additionally, we noticed that the Mpro-Hyperoside complex and Mpro-Remdesivir share interactions with regard to residues Ser144, His41, and Met49.

In addition, the binding energy was calculated; the compound exhibiting the highest ligand–target affinity had the lowest energy. Table 1 provides an overview of the various energetic features and physicochemical attributes of the interactions between the ligand and the protein.

Table 1 lists the bond energies for each molecule in ascending order and shows which residues are most frequently found in the active site when the Mpro protein docks with each ligand. Based on the obtained results, the reference molecule Remdesivir had an interaction energy of −7.70 kcal/mol, while hyperoside and quercitrin were the most effective inhibitors with an interaction energy of −8.8 kcal/mol. Thus, it is reasonable to assume that these two molecules are what could be triggering the ethyl acetate or *n*-butanol extract of Algerian fir leaves to exhibit antiviral properties.

#### 2.1.2. In Silico Antibacterial Effect Evaluation

The antibacterial effect of ethyl acetate and *n* butanol fractions obtained from the leaves of Algerian fir was examined by Benouchenne et al. [9] and Benouchenne et al. [19]. The results concluded that both extracts demonstrated a strong antibacterial ability against all tested bacterial strains. According to these findings, our in-silico work is in the context of identifying which molecule or compounds are responsible for the antibacterial effect, and which will be a promoting active ingredient of a natural antibiotic development. Actually, with the evolution of computer tools, it has become easy and helpful as well as reducing time and budget consumption. The in silico antibacterial activity evaluation of 12 molecules present in the extracts and the determination of the molecule responsible for this activity were carried out in the case of a Gram-negative bacterial strain (*Escherichia coli*) using as a reference the antibiotic tigecycline, and in the case of a Gram-positive bacterial strain (*Staphylococcus aureus*) using the antibiotic penicillin as a reference.

##### *Escherichia coli* 

The docking of 13 ligands (the 12 molecules of phenolic compounds and the reference antibiotic) was carried out with the receptor of the bacterium *Escherichia coli*, which is the 4PRV protein to determine the molecule responsible for the antibacterial activity of this extract in the case of Gram-negative bacteria. The program used was the DockThor Server (Table S2).

**Figure 4:**
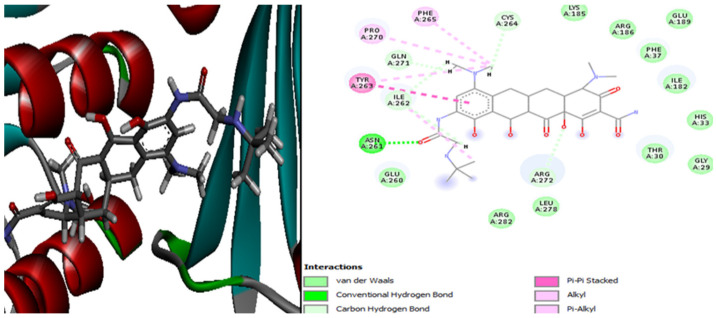
The positioning of the antibiotic tigecycline and intermolecular interactions within the complex with the *E. coli* 4PRV protein receptor.

Figure 4 presents the molecular interactions between the antibiotic tigecycline and the *E. coli* 4PRV protein receptor. The active site residues of the *E. coli* 4PRV protein that interacted with the tigecycline ligand are Arg272, Phe265, Pro270, Tyr263, Cys264, Ile262, Asn261, and Gln271.

**Figure 5:**
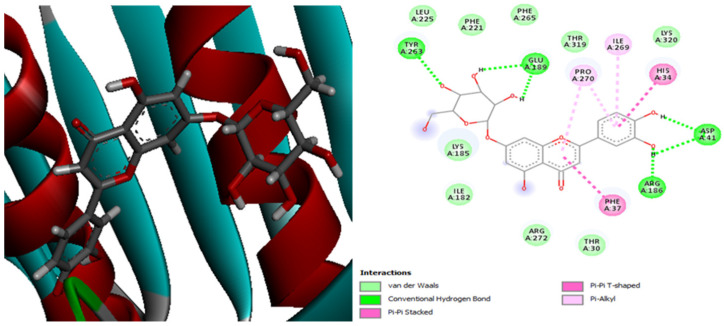
The positioning of luteolin-7-glucoside and intermolecular interactions within the complex with the *E. coli* 4PRV protein receptor.

Additionally, Figure 5 demonstrates that, with 11 residue interactions, namely, Tyr263, Glu189, Pro270, Ile269, His34, Asp41, Arg186, and Phe37, the complex 4PRV-luteolin-7-glucoside is the most stable.

Furthermore, the interaction energy was estimated. The results are presented in Table 2. It can be seen that luteolin-7-glucoside is the best inhibitor with the lowest interaction energy, −9.109 kcal/mol, compared to the reference product (antibiotic tigecycline), which presents an interaction energy equal to −9.027 kcal/mol. In this case, we can predict that luteolin-7-glucoside may be the molecule responsible for the antibacterial activity of the ethyl acetate and *n*-butanol fractions on *E. coli* bacteria.

##### *Staphylococcus aureus* 

**Figure 6:**
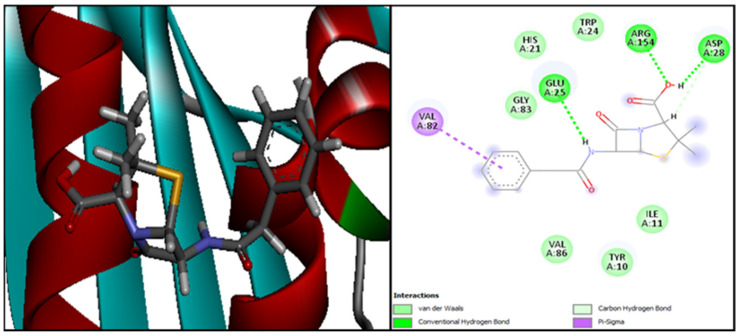
The positioning of penicillin and intermolecular interactions within the complex with the *S. aureus* 4URO protein receptor.

The interaction of the ligands with the 4URO are presented in Table S3. Figure 6 illustrates the outcomes of the molecular interactions between penicillin and the *S. aureus* 4URO protein.

Table S3 shows the interactions between the ligands and the *S. aureus* 4URO protein receptor. The Gram-positive bacteria *S. aureus*’s active site residues at the 4URO receptor that interacted with the penicillin ligand are Val82, Glu25, Arg154, and Asp28 (Figure 6). The following table (Table 3) provides an overview of the various energetic features and physicochemical aspects of the interactions between the ligand and the protein.

In order to identify the molecule causing the antibacterial activity in the case of Gram-positive bacteria, the 4URO receptor of the bacterium *Staphylococcus aureus* is docked with 13 ligands (penicillin and 12 phenolic compounds) using the Dockthor server. The results are summed up in the table above. According to these findings, hesperidin interacts with the bacterial receptor the best, having the lowest interaction energy (−7.941 kcal/mol) in comparison to penicillin (−6.704 kcal/mol). Thus, we can hypothesize that hesperidin may be the compound causing the ethyl acetate extract’s antibacterial effect on Gram-positive bacteria (Table 3).

Twelve bonds involving the residues Arg59, Arg100, Pro62, Glu33, Ile61, Gly60, Asp56, and Asp32 make up the most stable complex of 4URO-hesperidin (Figure 6). In this instance, we observe that the two molecules’ molecular interactions with the S. aureus 4URO protein receptor—hesperidin and the reference antibiotic penicillin—are distinct and do not share any residues in the complexes 4URO-penicillin and 4URO-hesperidin.

## 3. Discussion

This study is the first to identify the specific compounds responsible for the in silico antiviral and antibacterial activities of molecules found in the ethyl acetate and *n*-butanol fractions extracted from Algerian fir [9,19]. Molecular docking has become a major tool in computer-aided drug development. By searching through enormous pharmaceutical libraries for potential drug candidates, this innovative method can drastically cut down on the amount of energy, money, and time needed for drug discovery [20]. In the current work, we screened the inhibitory effects of the polyphenolic components of LC–MS/MS using a molecular docking technique.

Globally, the SARS-CoV-2 virus has a devastating impact on both human lives and economics. The virus continues to develop and take on new strains despite the availability of numerous treatment medications and vaccinations. Concerning viral variations have the potential to develop a dangerous resistance to current medications and vaccines. The identification of anti-SARS-CoV-2 medications from medicinal plants is facilitated by the use of machine learning in drug development [21].

The majority of the anticipated anti-SARS-CoV-2 compounds belong to polyphenols, such as the flavonoids category, accounting for 38.6% (390 compounds) [21]. Additionally, earlier research showed that flavonoids possess potent antiviral properties [22,23]. Within the flavonoid class, MOL000098 (quercetin) and MOL002008 (myricetin) can likewise limit SARS-CoV-2-2′s ability to infect by focusing on its 3CLpro [24,25]. Other groups, like aporphines [26], coumarins [27], and isoflavonoids [28], also possess antiviral qualities. The antiviral qualities of these types of chemicals could be useful in the fight against the COVID-19 pandemic.

Our findings demonstrate the strong antiviral activity of flavonoid molecules, especially hyperoside and quercitrin, which have an energy of −8.8 kcal/mol compared to the energy of −7.70 kcal/mol of Remdesivir, and show strong interactions and good binding affinity with the virus’s main protease. They are the most quantitatively abundant molecules in the extract, which is worth mentioning. Maaroufi et al. [29] reported that the extracts from *Abies sachalinensis* showed a stronger virucidal activity; the extracts from *Abies sachalinensis* contained nonvolatile flavonoids, mainly procyanidin- and prodelphinidin-type condensed tannin, and this may probably explain their rapid and potent virucidal activity against multiple viruses, including SARS-CoV-2 [30].

Strong redox substances that easily interact with proteins are polyphenols and quinones [31,32,33]. Protein structure can be altered, and cross-linked protein aggregates, such as membrane proteins, can develop as a result of protein interaction with polyphenols and quinones [32,33,34,35]. Maaroufi et al. [29] also reported that the extracts from *Abies sachalinensis* led to a significant viral titer reduction in SARS-CoV-2 ancestral and variant strains, affected the viral S and N proteins, and disrupted the SARS-CoV-2 genome. The extracts from *Abies sachalinensis* also induced envelope disruption in Beta coronavirus virions. Lipid vesicles rupture as a result of flavonoids like flavan-3-ols, which are the constituents of condensed tannins, causing phospholipid aggregation inside lipid membranes and consequent membrane stiffness [36]. Quinones have also been shown to breach the viral envelope [37] and pierce lipid bilayers, altering their biophysical characteristics [38].

Another problem that humans face are bacterial infections. For that raison, plants are now one of the most important sources for identifying a new bioactive chemical to treat human diseases caused by pathogenic bacteria [39,40,41]. According to Brantner et al. [42], at certain concentrations, phenolic chemicals, flavonoids, and steroids may prevent the growth of bacteria. Important antibacterial agents include polyphenols like tannins and flavonoids such as myricetin, epigallocatechin, catechin, quercetin, and luteolin [43]. Also, anthraquinones and dihydroxyanthraquinone such as saponins have direct antimicrobial activity [44,45,46].

Based on our findings, the antibacterial activity against Gram-negative *Escherichia coli* is attributed to the Luteolin-7-glucoside molecule. This molecule exhibits strong interactions with the 4PRV protein of these bacteria and has the lowest energy at −9.109 kcal/mol, in comparison to the antibiotic Tigecycline, which has an energy of −9.027 kcal/mol. When compared to the reference molecule, which has an interaction energy of −6.704 kcal/mol, the majority of molecules for Gram-positive *Staphylococcus aureus* showed stronger interactions with the 4URO protein and better antibacterial activity than penicillin. One such molecule is likely hesperidin, which exhibited the lowest energy of all molecules at −7.941 kcal/mol. The results obtained in this study proved that the presence of weak binding interactions can activate specific biological responses in proteins, such as inhibition through specific domains. These results are in agreement with the reported pattern by Abdelli et al. [47].

## 4. Materials and Methods

### 4.1. Ligand Preparation

In 2020 and 2021, Benouchenne and her colleagues [9,19] determined the chemical composition of ethyl acetate and *n*-butanol fraction obtained from Algerian fir leaves. The results are summarized in Table 4. The extraction process was realized by means of cold maceration using methanol as solvent (80%, *v*/*v*). The obtained crude extract was fractionated using several solvents with increased polarities, starting with dichloromethane, followed by ethyl acetate and n-butanol. Later fractions were subjected to LC-MS/MS analysis.

### 4.2. Programs and Methods

Molecular mechanic computations were performed using Hyperchem™ 8.0.10 software (Hypercube Inc., Gainesville, FL, USA). Avogadro Version 1.1.1 was used; it is an open-source molecular builder and visualization tool (http://avogadro.cc/, accessed on 15th March 2022) with a number of tools designed for molecular modeling that enables the editing and visualization of molecular models. MOPAC^®^ (Molecular Orbital PACkage) computes force constants for molecules, radicals, ions, and polymers as well as quantities. COVID-19 Docking Server [48,49] was employed to forecast binding patterns between COVID-19 targets and ligands, such as small molecules, and for molecular visualization (https://ncov.schanglab.org.cn, accessed on 30th June 2020). Dockthor server [50,51] gave the basic instructions for getting the ligand and protein ready, allowing us to alter the residues’ protonation states and specify the ligand’s degree of flexibility. BIOVIA Discovery Studio Visualizer [52] allowed the visualization of biomolecular structures and sequences.

### 4.3. Antiviral Effect

#### Main Protease of SARS-CoV-2 Virus (Mpro)

The main protease of the SARS-CoV-2 virus is thought to be a key player in the pathogenicity of SARS-CoV-2, and was chosen as the molecular target based on a review of the literature. Because it is involved in the viral entry into the host [51], given the pivotal role of Mpro in the viral life cycle, it becomes an attractive target for the design of anti-SARS drugs. The three-dimensional crystal structures of the main protease of the SARS-CoV-2 virus were downloaded from the Research Collaboratory Structural Bioinformatics-Protein Data Bank (RCSB-PDB) [52]. The PDB code used was 6W63.

### 4.4. Antibacterial Effect

#### 4.4.1. PRV for *Escherichia coli* 

A complex of 43-kDa N-terminal fragment of *E. coli* GyrB with ADP was used; the 3D structure was downloaded from RCSB PDB [52] as a pdb file. The PDB code used was 4PRV.

##### 4.4.2. URO for *Staphylococcus aureus* 

It consists of novobiocin combined with the 24 kDa N-terminal domains of *Staphylococcus aureus* gyrase B. From RCSB PDB [52], the 3D structure as a pdb file was downloaded, and we only worked on chain A. The PDB code used was 4URO.

## 5. Conclusions

The conclusion of this study is that the polyphenolic compound of *Abies numidica* De Lannoy has potential as an antiviral against the Mpro enzyme of the SARS-CoV-2 virus, which is predicted in silico. Based on the molecular docking data, hyperoside and quercitrin (−8.8 kcal/mol) have greater SARS-COV-2 antiviral activity than Remdesivir (−7.70 kcal/mo). The findings reveal that luteolin-7-glucoside and hesperidin may be further explored as antibacterial agents. Therefore, it would be convenient to extract these biomolecules specifically from Numidian fir leaves and test them again in vivo to confirm their impact on bacteria and the coronavirus. And why not create natural antiviral and antibacterial drugs that have no adverse effects on human health compared to their synthetic counterparts?

## Figures and Tables

**Table 1:** Energy characteristics and interactions between the ligands and the Mpro receptor.

Molecules	Score (kcal/mol)	Met A: 165	His A: 41	Met A: 49	Leu A: 141	His A: 164	Gln A: 189	His A: 163	Glu A: 192	Leu A: 167	His A: 172	Leu A: 27
Hyperoside	−8.8	+	+	+	+	+	-	-	-	-	-	-
Quercitrin	−8.8	+	+	+	-	+	-	+	-	-	-	-
Astragalin	−8.6	-	+ +	+ +	-	-	-	+	-	-	-	-
Rutin	−8.4	+	+++	+	-	-	-	-	-	-	-	-
Hesperidin	−8.2	+	-	-	-	+	-	-	+ +	+	-	-
Apigetrin	−8	+	-	-	+	-	+	-	-	-	-	-
Quercetin	−8	+	+	+	+	+	+	-	-	-	-	-
Apigenin	−7.9	+	+	+	+	+	-	+	-	-	-	-
Luteolin	−7.9	+	+	+	+	+	-	-	-	-	-	-
Luteolin-7-glucoside	−7.9	+ +	-	-	-	-	+ +	+	-	-	-	-
Remdesivir	−7.70	++	+++	+	-	-	+	-	-	-	-	-
Chlorogenic acid	−7.4	+	-	-	+	-	-	-	-	-	-	-
Protocatechuic acid	−5.3	-	-	-	+	-	-	-	-	-	-	-

+: Pi-Cation; +: Unfavorable Acceptor-Acceptor; +: Pi-Donor Hydrogen Bond; +: Pi-Sulfur; +: Conventional Hydrogen Bond; +: Sulfur-X; +: Pi-Alkyl.

**Table 2:** Energy characteristics and interactions between the ligands and *E. coli* 4PRV protein receptor.

Compounds	Score (kcal/mol)	Glu A: 189	ArgA: 272	Lys A: 185	Arg A: 186	Ile A: 182	His A: 34	Phe A: 37	Thr A: 319	Thr A: 30	Phe A: 265	Phe A: 221
Luteolin-7-glucoside	−9.109	++	-	-	+	-	+	+	-	-	-	-
Tigecycline	−9.027	-	+	-	-	-	-	-	-	-	+	-
Rutin	−8.547	++ +	+	-	-	-	-	-	-	-	-	-
Apigetrin	−8.483	++	+ +	-	-	-	-	-	-	-	-	-
Hyperoside	−8.451	+	-	-	-	-	-	-	-	-	-	-
Hesperidin	−8.443	++	-	++++	-	-	-	-	-	-	-	-
Astragalin	−7.890	+	+	+	-	-	-	-	-	+	-	-
Quercitrin	−7.765	+	+ +	-	-	+	-	-	+	-	-	-
Luteolin	−7.656	+	+ +	+	-	-	-	+	-	-	-	-
Quercetin	−7.632	+ +	+	-	+	+	-	-	-	-	-	-
Apigenin	−7.614	+ +	+	-	+	+	+	-	-	-	-	-
Chlorogenic Acid	−7.544	+	+	+	-	-	-	-	-	-	-	-
Protocatechuic Acid	−7.030	-	-	-	+	-	+	-	+	-	-	-

+: Pi-Cation; +: Unfavorable Acceptor-Acceptor; +: Pi-Donor Hydrogen Bond; +: Pi-Sulfur; +: Pi-Sigma; +: Conventional Hydrogen Bond; +: Sulfur-X; +: Pi-Alkyl.

**Table 3:** Energy characteristics and interactions between the ligands and *S. aureus* 4URO protein receptor.

Compounds	Score (kcal/mol)	Arg A: 154	Glu A: 25	Asp A: 32	Ile A: 61	Arg A: 59	Gly A: 81	Glu A: 33	Gly A: 83	Ile A: 11	Gly A: 58	Ile A: 77
Hesperidin	−7.941	-	-	-	++	+ + +	-	+	-	-	-	-
Luteolin-7-glucoside	−7.681	+ +	+	-	-	-	-	-	-	+	-	-
Quercetin	−7.678	+ +	-	-	-	-	-	-	-	-	-	-
Apigetrin	−7.645	-	+ +	++	+	-	++	-	+	-	-	-
Quercitrin	−7.598	-	-	+	++	+	-	+	-	-	-	-
Hyperoside	−7.473	+ +	++	-	-	-	-	-	-	-	-	-
Apigenin	−7.471	-	-	+	++	-	-	+	-	-	-	-
Chlorogenic acid	−6.798	+	+	+ +	-	-	+	-	+	-	-	-
Penicillin	−6.704	+	+	-	-	-	-	-	-	-	-	-
Luteolin	−6.660	++	+	-	-	-	-	-	-	+	-	-
Astragalin	−6.587	-	-	-	-	-	-	-	-	-	-	-
Rutin	−6.340	+	+	+	-	-	-	-	-	-	-	-
Protocatechuic acid	−5.926	-	-	-	-	-	-	-	-	-	-	-

+: Pi-Cation; +: Pi-Donor Hydrogen Bond; +: Conventional Hydrogen Bond; +: Pi-Alkyl.

**Table 4:** Chemical composition of ethyl acetate and *n*-butanol fractions obtained from Algerian fir needles by means of LC-MS/MS analysis.

No.	Analytes	RT ^a^	Precursor ion (m/z) ^b^	Fragmentations	Ionization mode	Equation	^c^ R^2^	U ^f^	*n*-BuOH (µg/g)	EAF (µg/g)
1	Protocatechuic acid	7.00	153.4	109.0–108.0	Neg	y = 590.460x + 120.26	0.9909	0.0215	**N.D**	**71.62**
2	Chlorogenic acid	8.03	353.3	191.2–85.0	Neg	y = 697.935x + 87418.5	0.9910	0.0299	**9.66**	**15.59**
3	Luteolin-7-glucoside	13.20	447.0	285.1–284.1	Neg	y = 215.412x + 36852.1	0.9939	0.0086	**14.6**	**43.17**
4	Rutin	13.67	609.1	300.1–301.1	Neg	y = 469.333x + 30144.8	0.9902	0.0136	**102.62**	**27.58**
5	Hesperidin	13.68	611.1	303.0–449.3	Poz	y = 2539.52x + 123981	0.9942	0.0162	**42.02**	**7.83**
6	Hyperoside	13.69	463.0	300.1–271.0	Neg	y = 185.593x + 8126.67	0.9905	0.0126	**399.91**	**3370.96**
7	Apigetrin	14.54	431.0	268.1–269.1	Neg	y = 1052.01x + 146897	0.9902	0.0132	**23.19**	**192.56**
8	Quercitrin	14.98	447.0	300.0–301.1	Neg	y = 175.298x + 33626.6	0.9918	0.0133	**20.44**	**2300.33**
9	Astragalin	15.13	447.0	284.1–227.1	Neg	y = 329.506x + 44598.6	0.9900	0.0153	**147.22**	**3391.36**
10	Quercetin	17.10	301.2	151.1–179.1	Neg	y = 1826.89x − 146948	0.9962	0.0573	**N.D**	**24.75**
11	Luteolin	17.78	285.2	133.1–151.0	Neg	y = 3166.03x + 495252	0.9901	0.0188	**N.D**	**2.41**
12	Apigenin	19.20	269.2	117.0–151.1	Neg	y = 3115.89x + 483037	0.9910	0.0181	**N.D**	**6.64**
13	Pseudohypericin	26.34	519.0	487.1–475.1	Neg	y = 2548.96x + 468900	0.9908	0.0172	**N.D**	**N.D.**
14	Hyperforin	28.97	535.3	383.3–315.2	Neg	y = 44260.6x + 203394	0.9901	0.0418	**N.D**	**N.D.**
15	Hypericin	30.18	503.0	405.1–433.1	Neg	y = 7676.03x + 605593	0.9925	0.0189	**N.D**	**N.D.**

RT ^a^: Retention time, ^c^ R^2^: Correlation coefficient, U ^f^ (%): Percent relative uncertainty at 95% confidence level (k = 2), **N.D**: NOT detected, **EAF**: ethylacetate fraction, ***n*-BuOH**: *n*-butanol fraction.

## Data Availability

The data presented in this study are available on request from the corresponding author.

## Supplementary Materials

The following supporting information can be downloaded at: https://www.mdpi.com/article/10.3390/plants13091246/s1. Table S1. Superposition and intermolecular interactions between the different ligand determined in Algerian fir and the main protease of SARS-COV-2 virus using PM7 Method (COVID-19); Table S2. Superposition and intermolecular interactions between the different ligand determined in Algerian fir and 4PRV presents in *E. coli* using PM7 Method; Table S3. Superposition and intermolecular interactions between the different ligand determined in Algerian fir and *S. aureus* 4URO protein receptor using PM7 Method.
