# Supplementary material for: The First Records of the In Silico Antiviral and Antibacterial Actions of Molecules Detected in Extracts of Algerian Fir (Abies numidica De Lannoy) Using LC-MS/MS Analysis
Source: Plants (Basel). 2024 Apr 30;13(9):1246. doi: 10.3390/plants13091246 (PMC11085235; doi:10.3390/plants13091246)
Supplement: Supplementary file 1 [file plants-13-01246-s001.zip › plants-2953752-Supplementary Material.pdf]

Table S1. Superposition and intermolecular interactions between the different ligand determined in Algerian fir and the main protease of Sars-cov 2 virus using PM7 Method (Covid19)

<table>
<tr><th>Compounds</th><th>Molecular Docking</th><th>H-Bond</th><th>Hydrophobic</th><th>Interactions</th></tr>
<tr><td>Apigenin</td><td>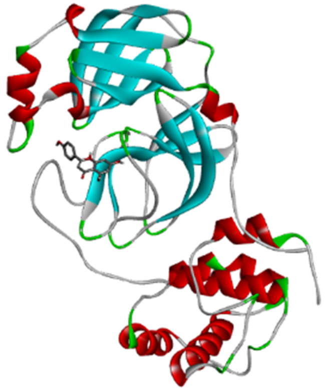</td><td>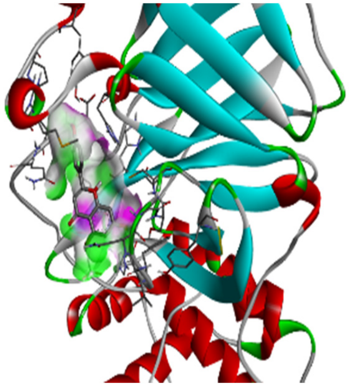</td><td>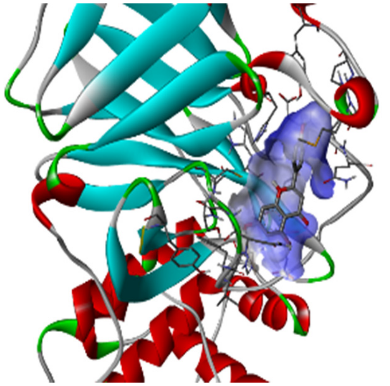</td><td>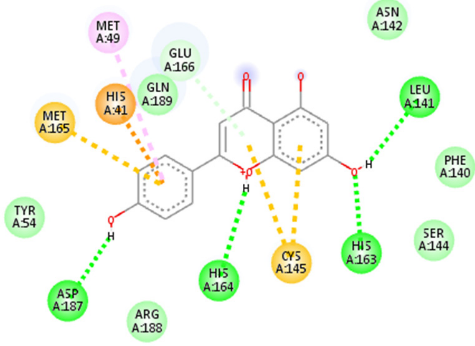
</td></tr>
<tr><td>Apigetrin</td><td>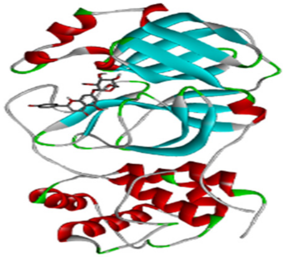</td><td>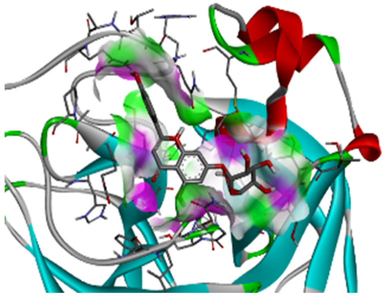</td><td>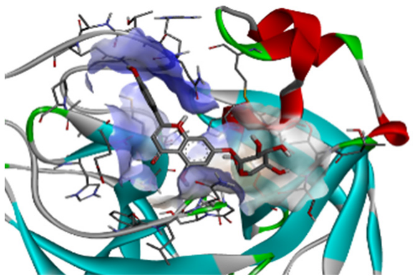</td><td>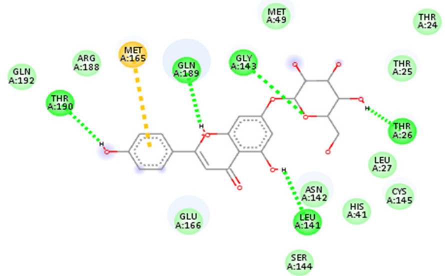
</td></tr>
</table>

Astragalin	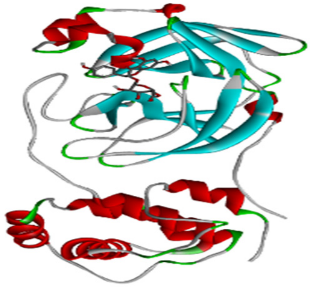	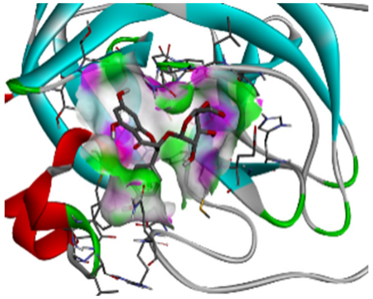	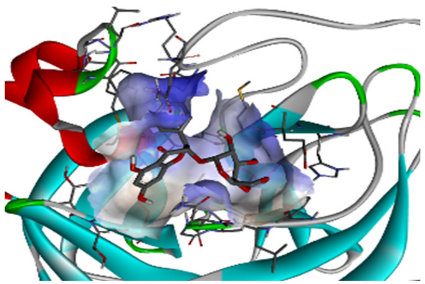	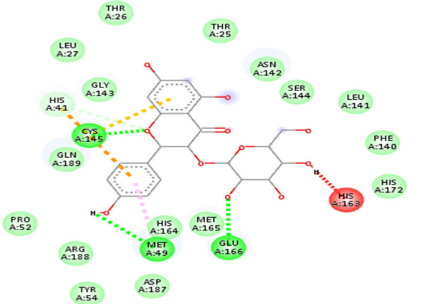
chlorogenic Acid	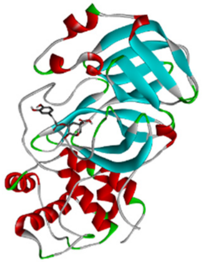	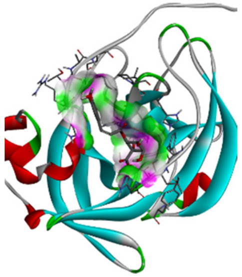	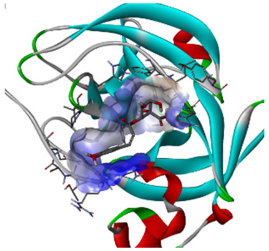	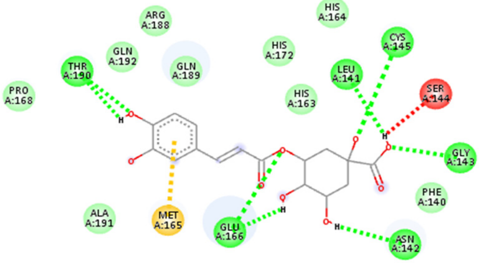
Hesperidin	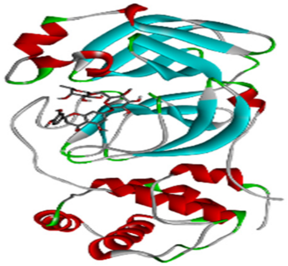	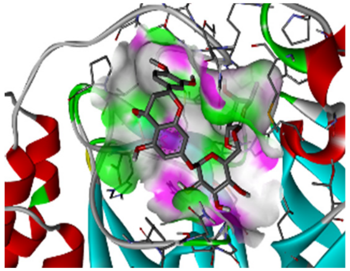	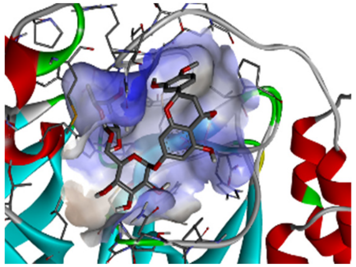	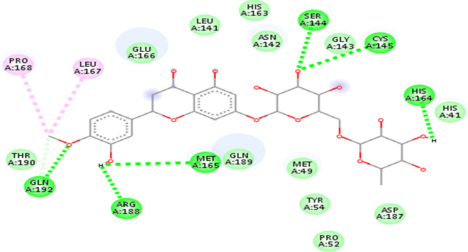

<table>
<tr><td>Hyperoside</td><td>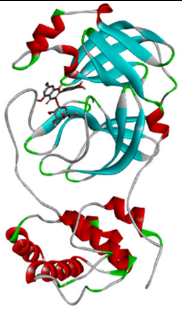</td><td>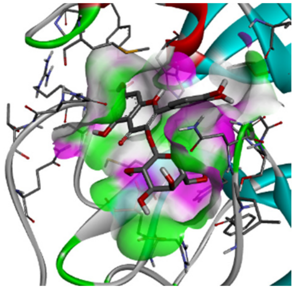</td><td>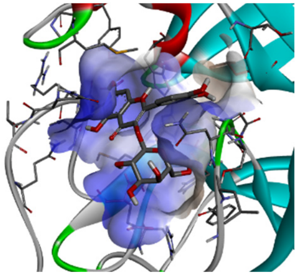</td><td>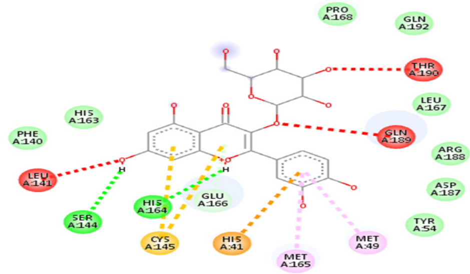
</td></tr>
<tr><td>Luteolin</td><td>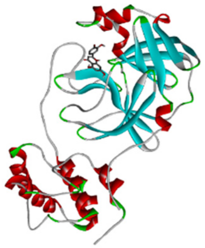</td><td>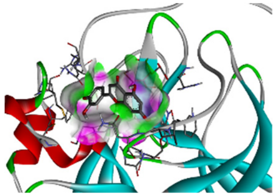</td><td>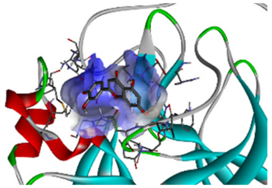</td><td>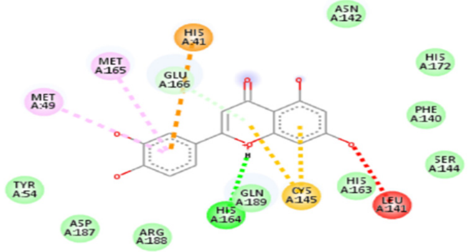
</td></tr>
<tr><td>Luteoline-7-glucoside</td><td>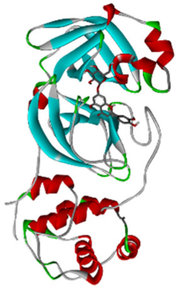</td><td>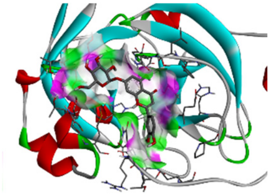</td><td>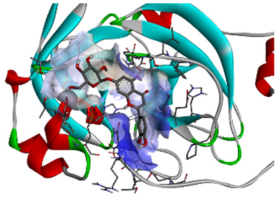</td><td>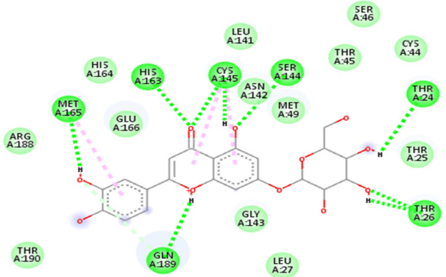
</td></tr>
</table>

Protocatechuic Acid	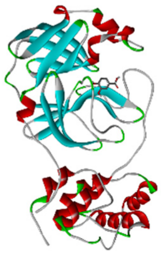	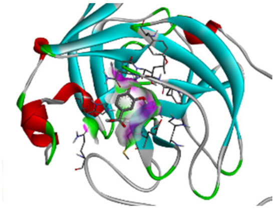	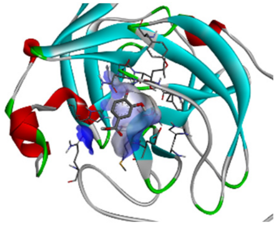	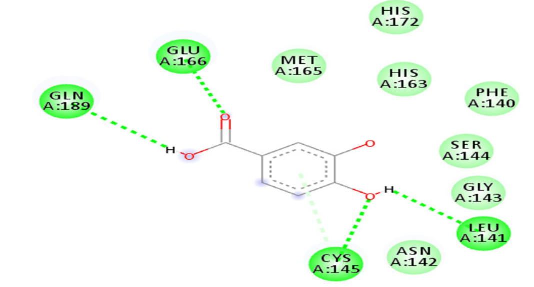
Quercetin	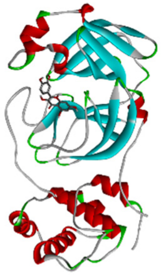	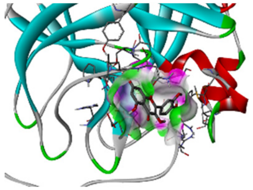	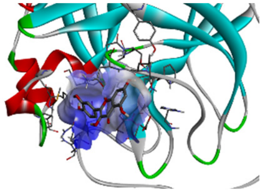	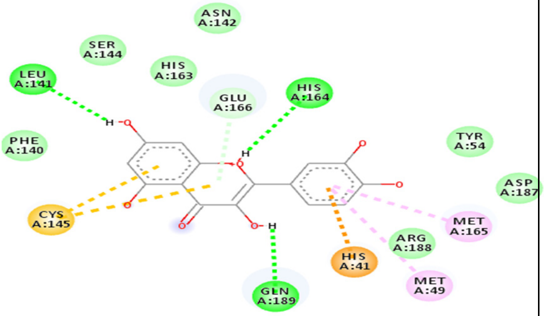
Quercitrin	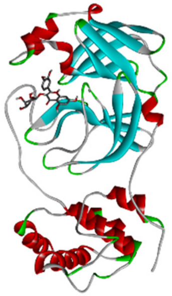	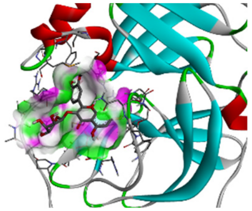	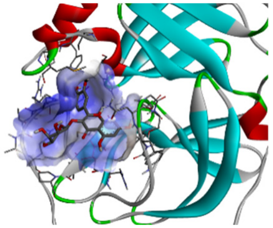	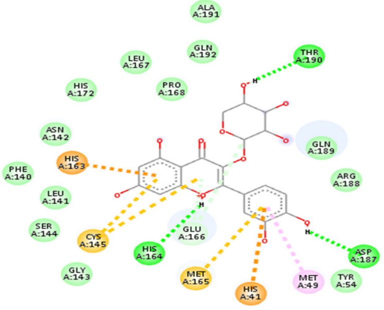

Rutin	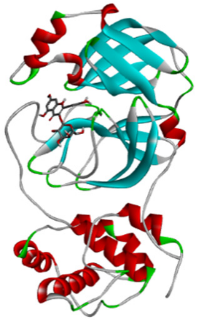	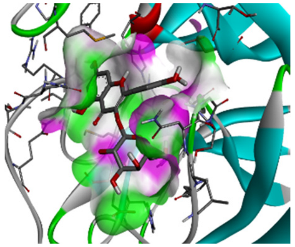	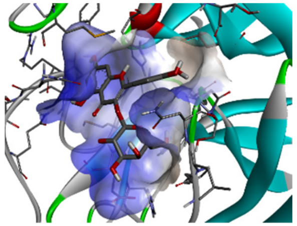	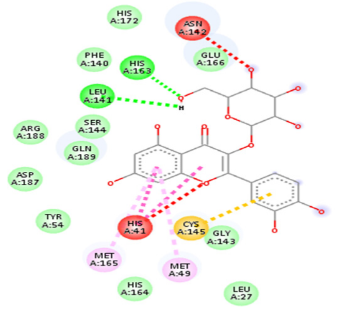
Redmisivir	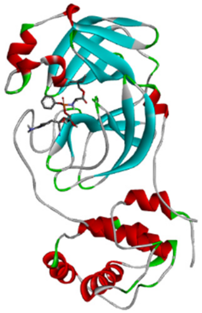	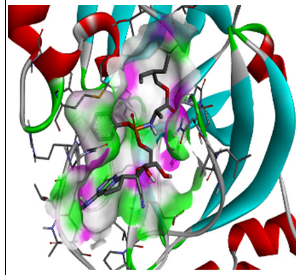	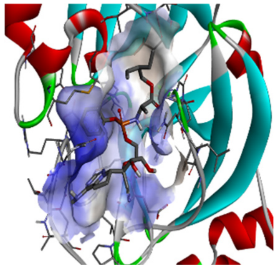	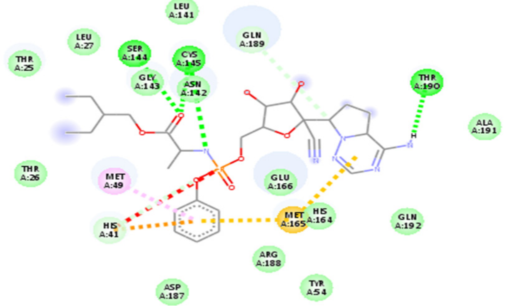

+: Van der Waals; +: Pi-Cation; +: Unfavorable Acceptor -Acceptor; +: Pi-Donor Hydrogen Bond; +: Pi-Sulfur; +: Pi-Sigma; +: Conventional Hydrogen Bond; +: Sulfur -X; +: Pi-Alkyl.

Table S2. Superposition and intermolecular interactions between the different ligand determined in Algerian fir and 4PRV presents in *E. coli* using PM7 Method

Molecule	Molecular Docking	H-Bond	Hydrophobic	Interactions
Apigenin	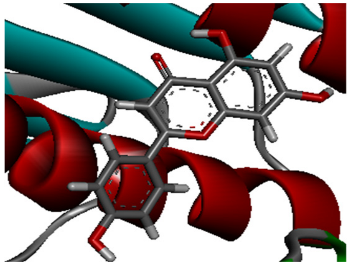	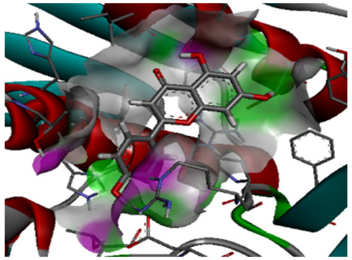	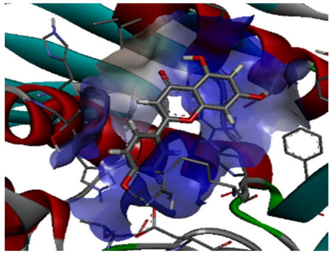	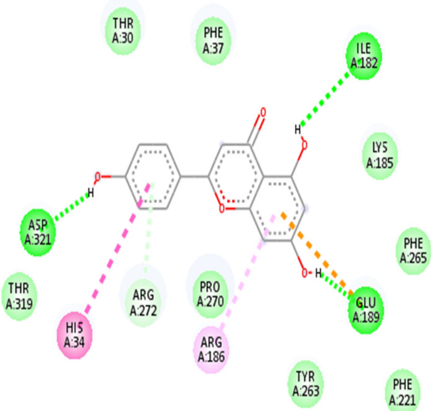

Apigetrin	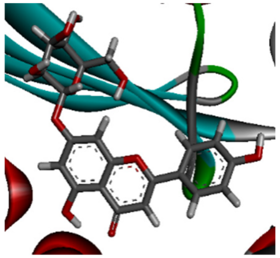	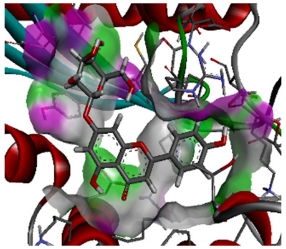	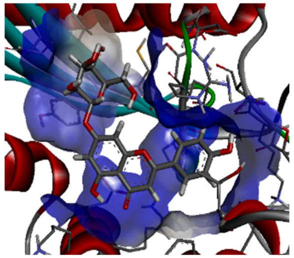	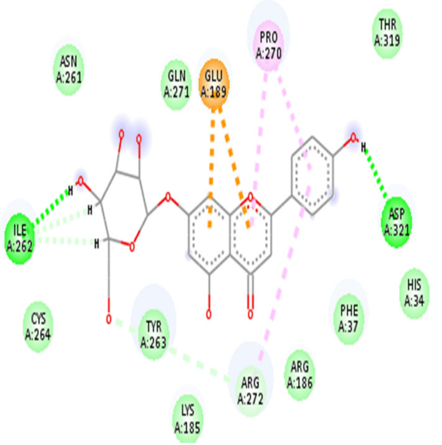
Astragalin	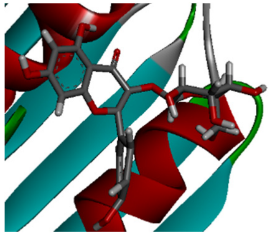	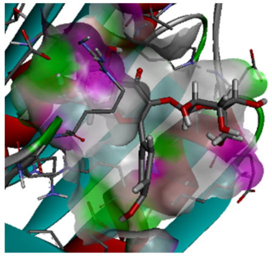	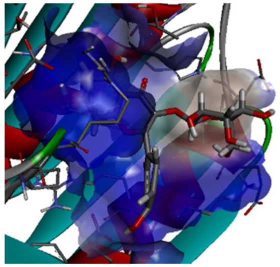	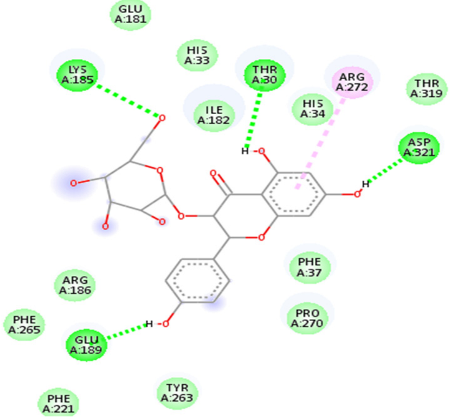

<table>
<tr><td>Chlorogenic Acid</td><td>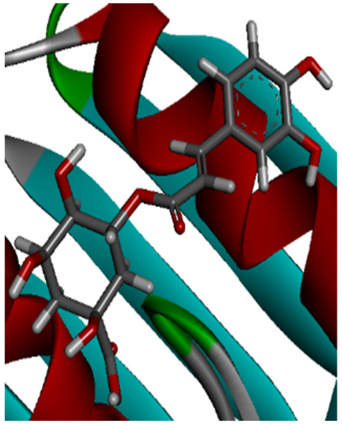</td><td>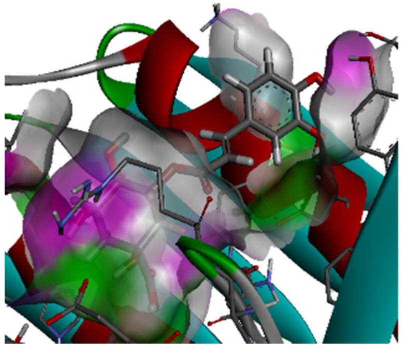</td><td>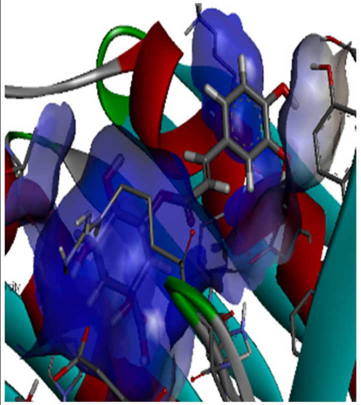</td><td>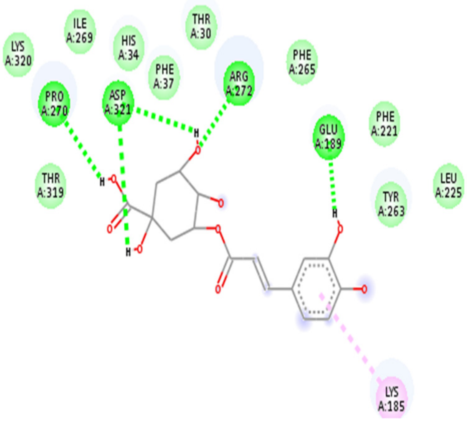
</td></tr>
<tr><td>Hesperidin</td><td>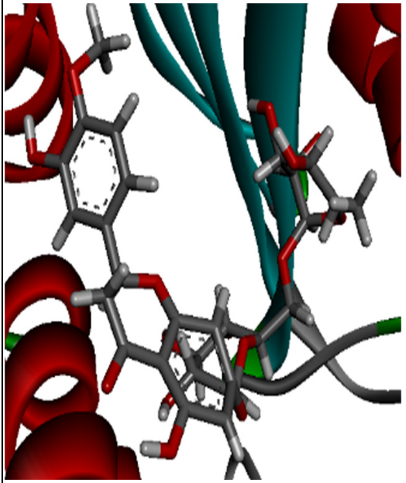</td><td>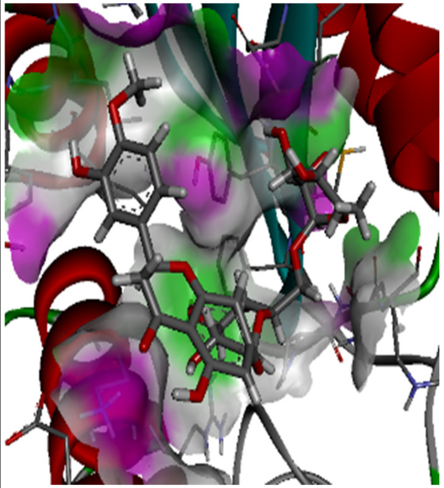</td><td>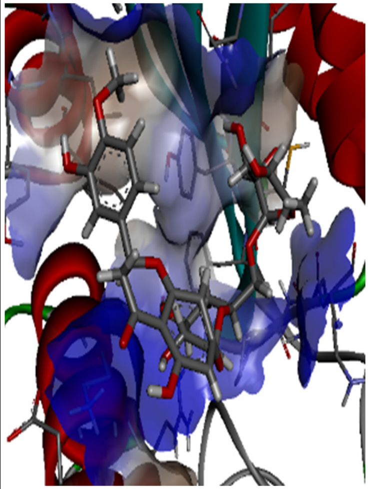</td><td>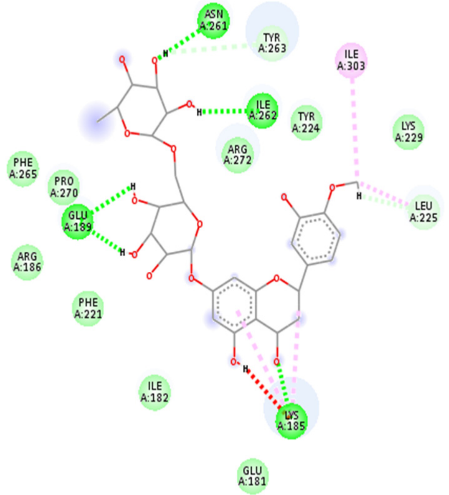
</td></tr>
</table>

Hyperoside	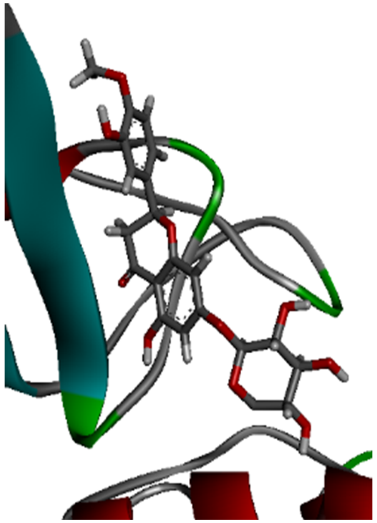	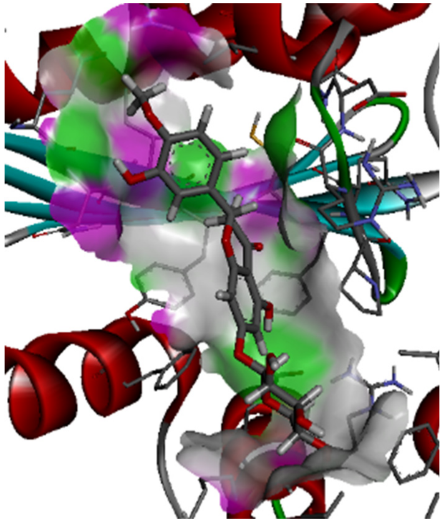	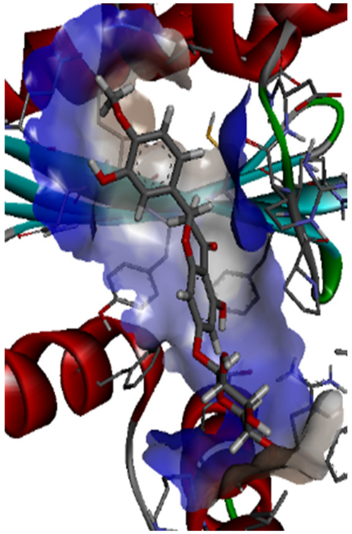	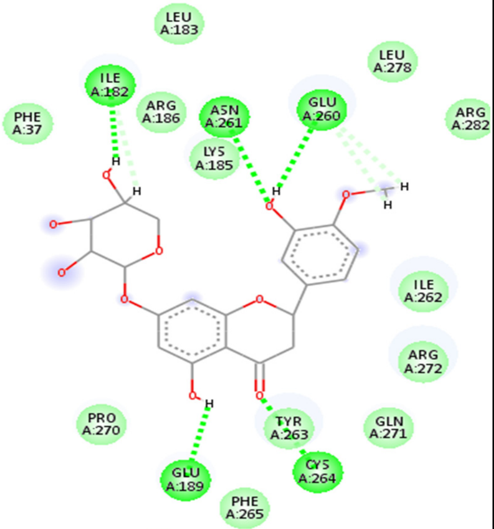
Luteolin	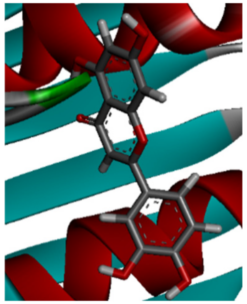	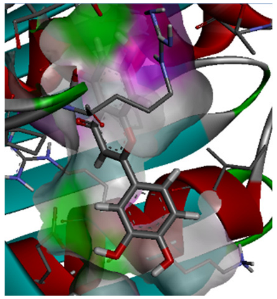	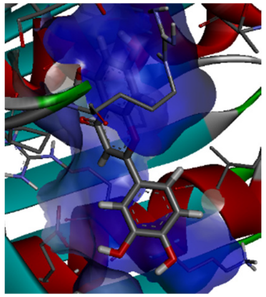	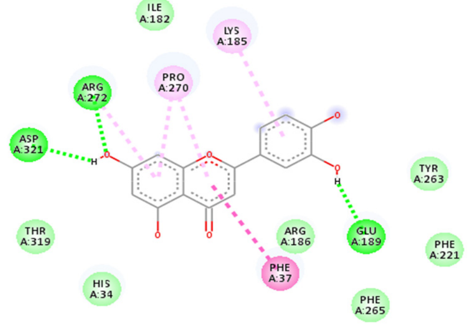

<table>
<tr>
<td>Luteoline-7-glucoside</td>
<td>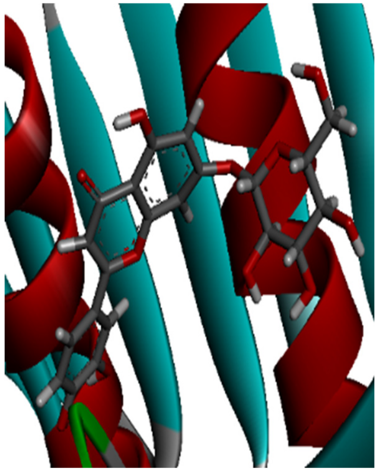</td>
<td>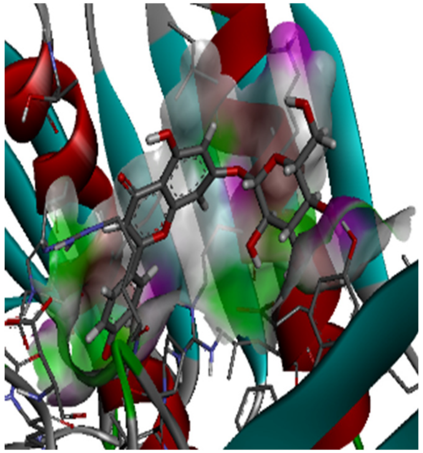</td>
<td>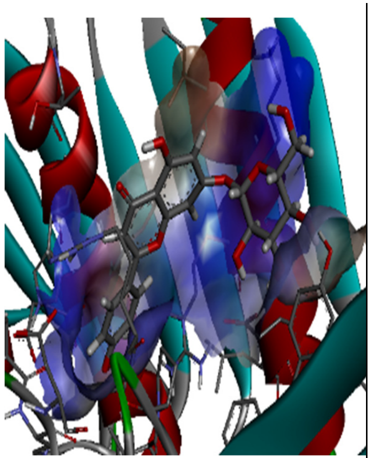</td>
<td>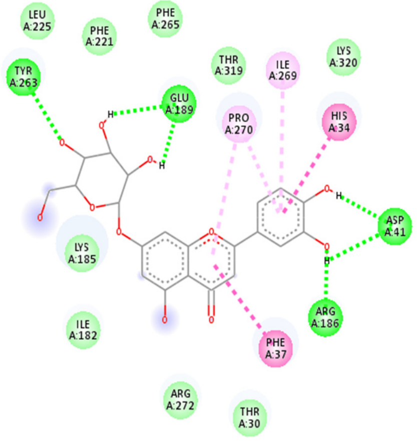
</td>
</tr>
<tr>
<td>Protocatechuic Acid</td>
<td>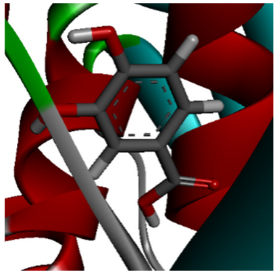</td>
<td>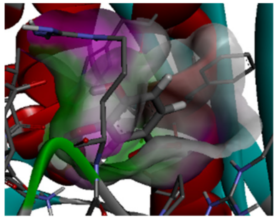</td>
<td>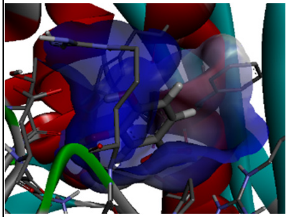</td>
<td>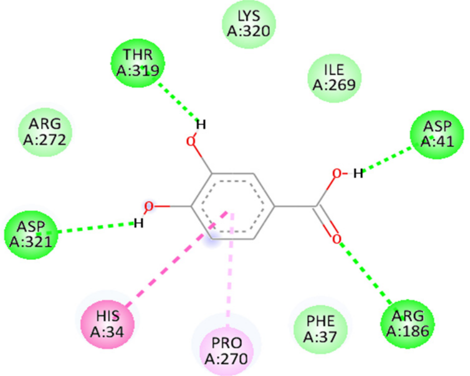
</td>
</tr>
</table>

Quercetin	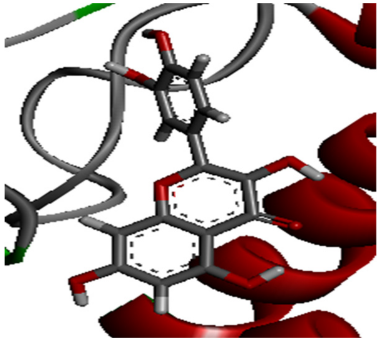	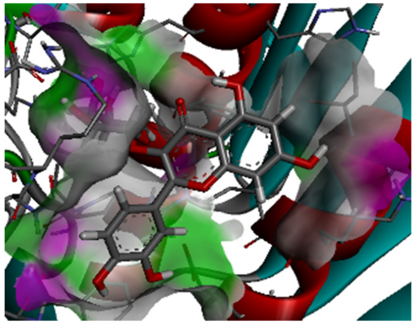	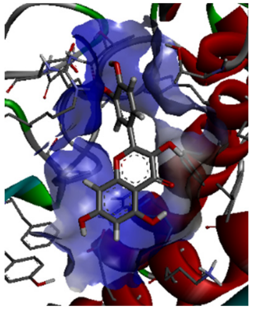	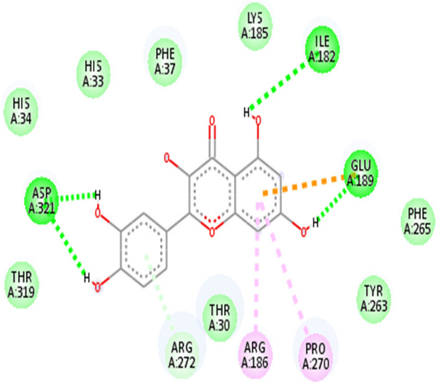
Quercitrin	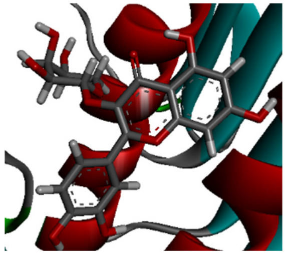	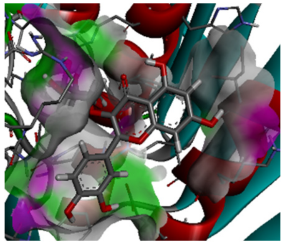	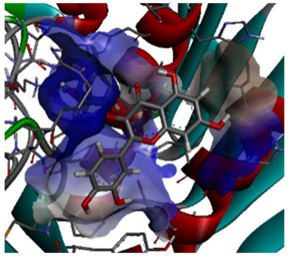	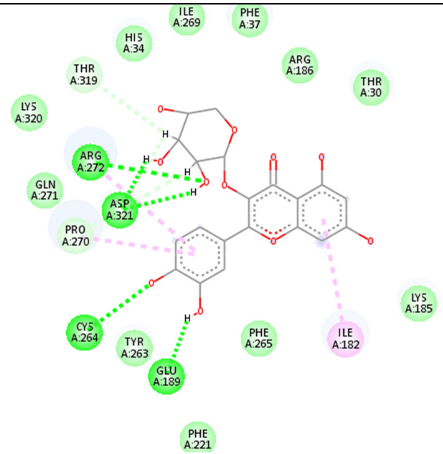

Rutin	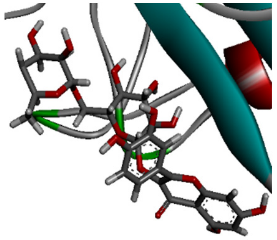	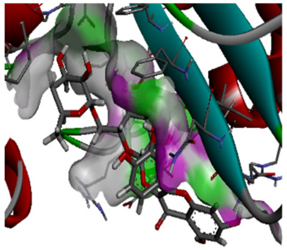	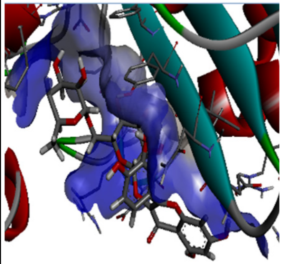	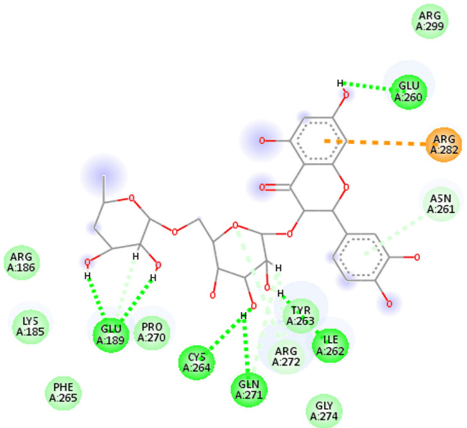
Tigecycline	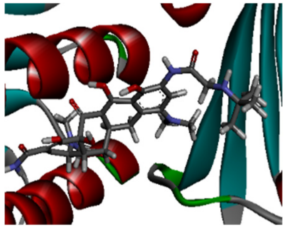	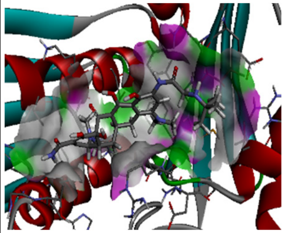	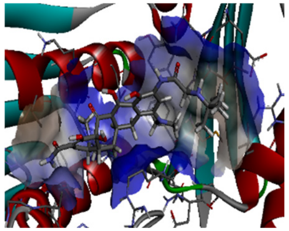	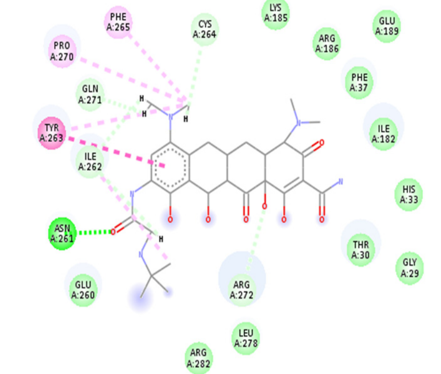

Table S3. Superposition and intermolecular interactions between the different ligand determined in Algerian fir and *S. aureus* 4URO protein receptor using PM7 Method

<table>
<tr><th>Molecule</th><th>Molecular Docking</th><th>H-Bond</th><th>Hydrophobic</th><th>Interactions</th></tr>
<tr><td>Apigenin</td><td>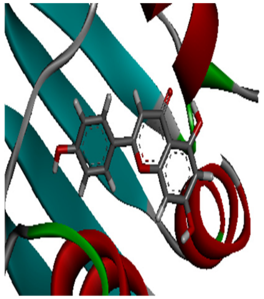</td><td>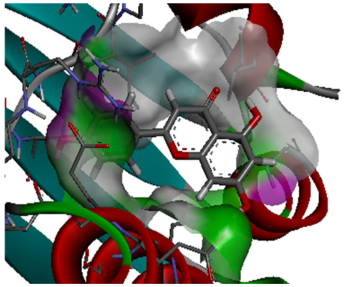</td><td>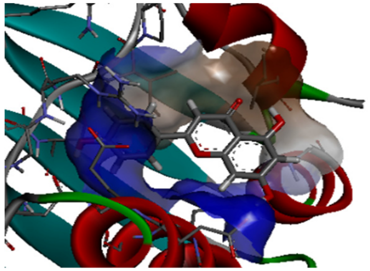</td><td>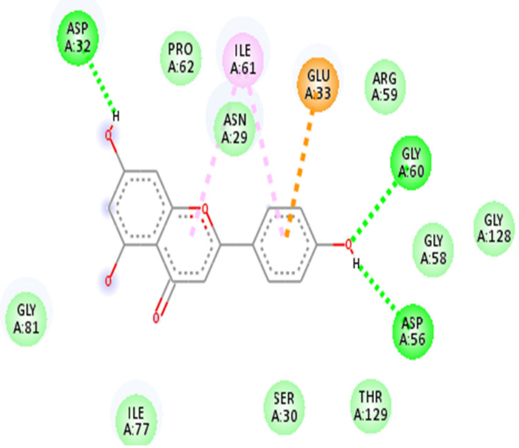
</td></tr>
<tr><td>Apigetrin</td><td>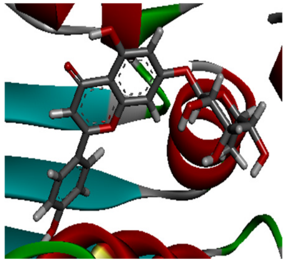</td><td>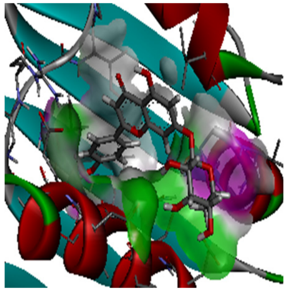</td><td>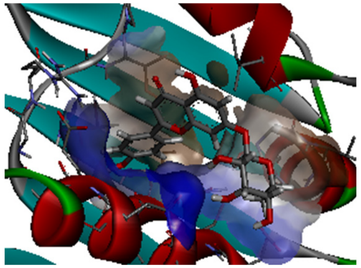</td><td>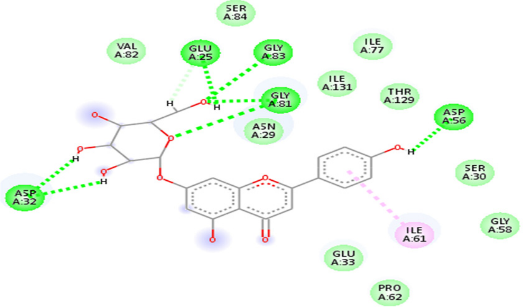
</td></tr>
</table>

Astragalin	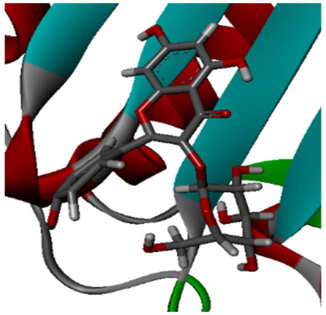	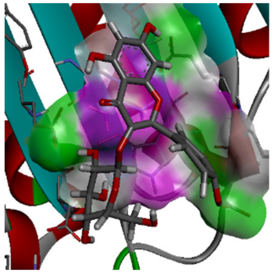	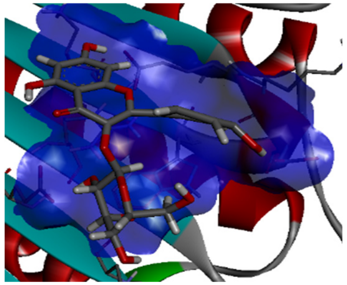	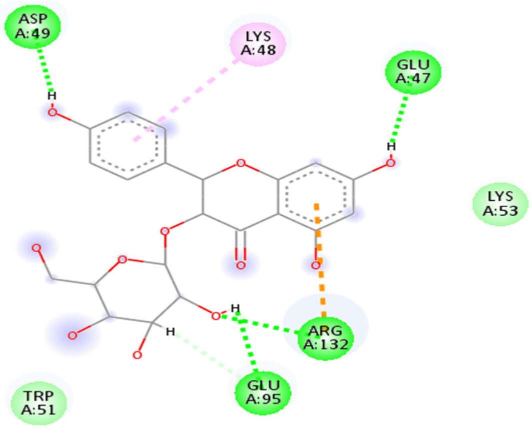
Chlorogenic Acid	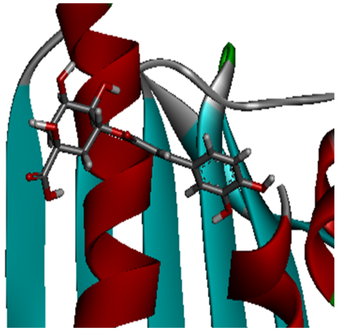	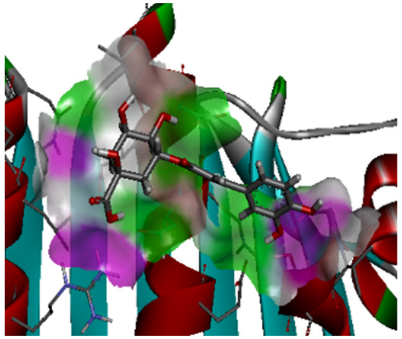	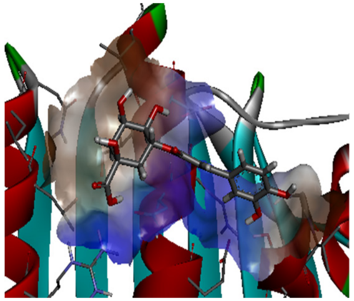	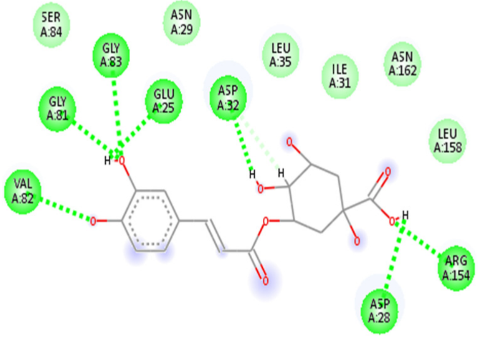

Hesperidin	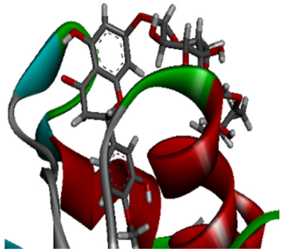	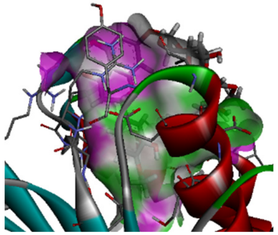	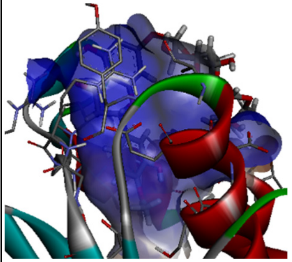	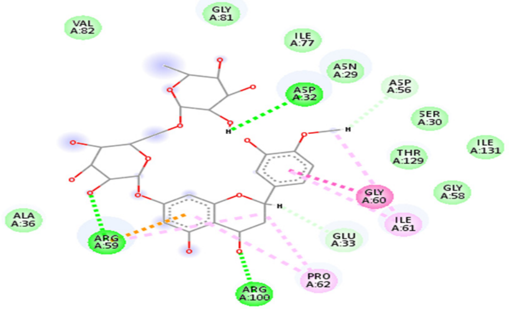
Hyperoside	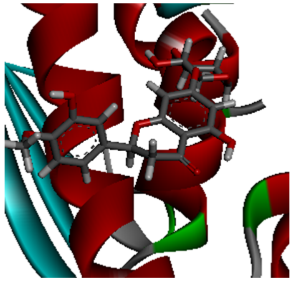	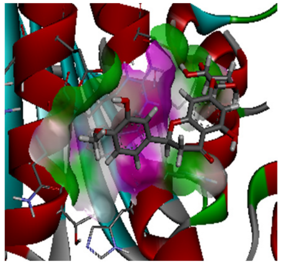	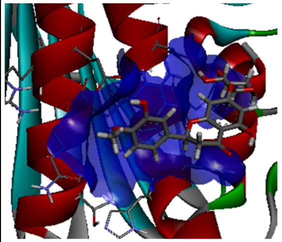	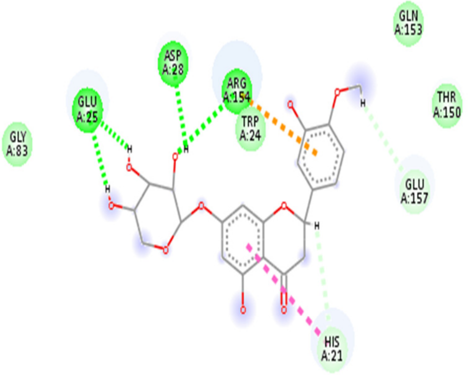
Luteolin	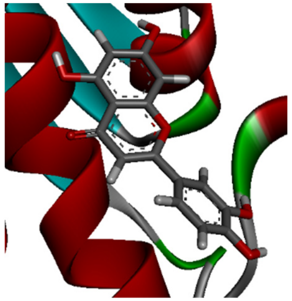	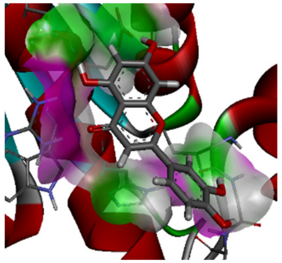	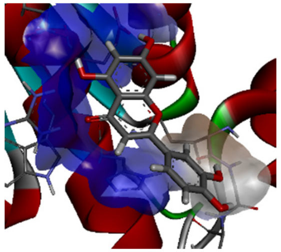	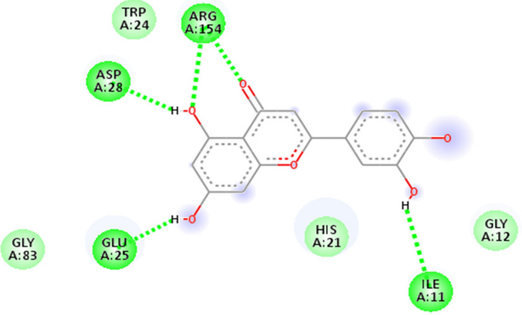

<table>
<tr><td>Luteoline-7-glucoside</td><td>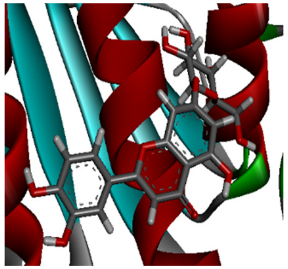</td><td>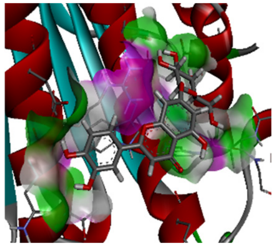</td><td>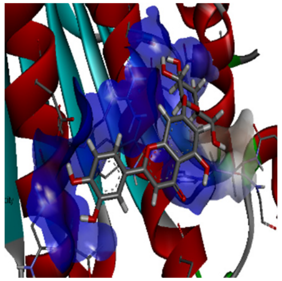</td><td>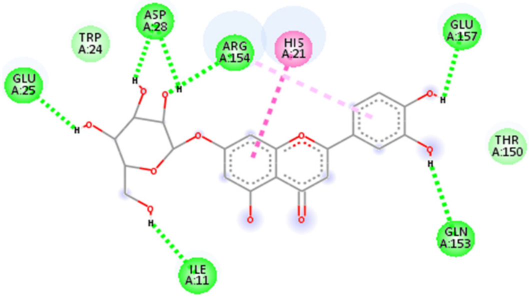
</td></tr>
<tr><td>Protocatechuic Acid</td><td>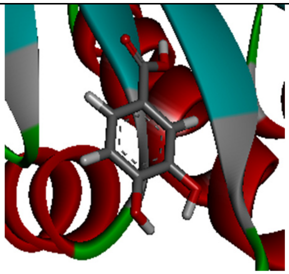</td><td>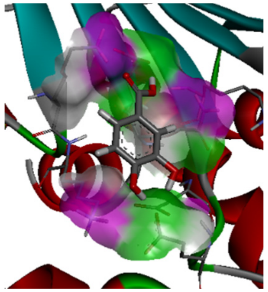</td><td>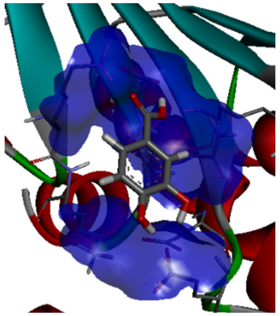</td><td>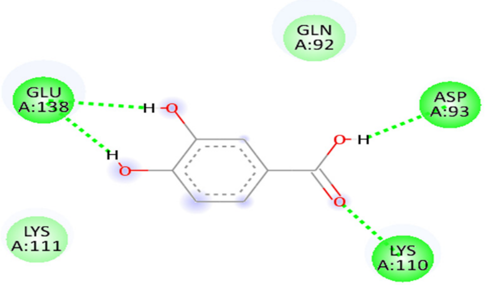
</td></tr>
<tr><td>Quercetin</td><td>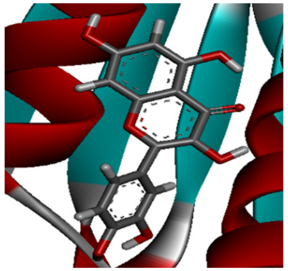</td><td>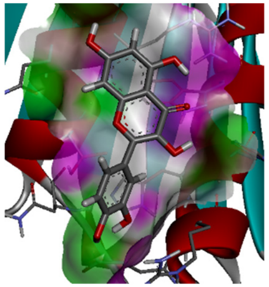</td><td>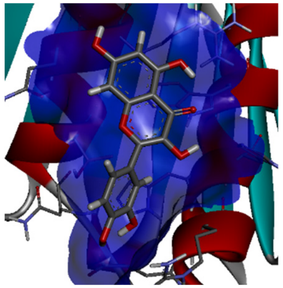</td><td>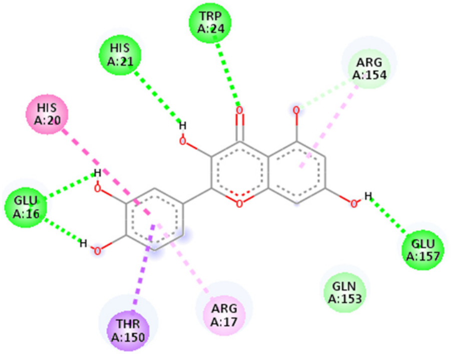
</td></tr>
</table>

Quercitrin	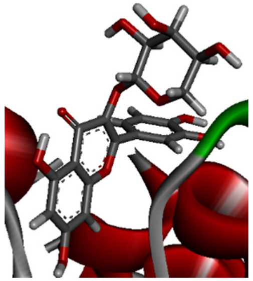	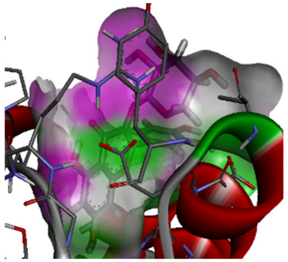	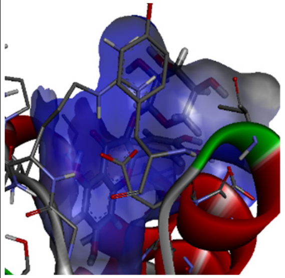	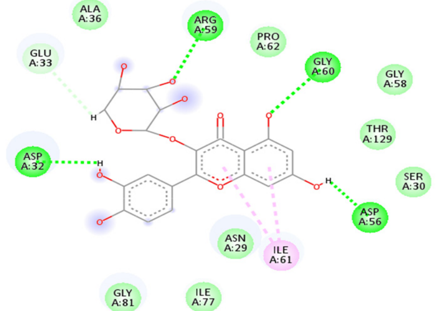
Rutin	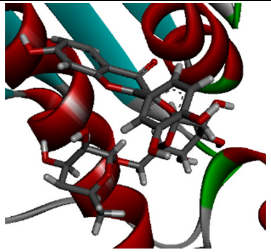	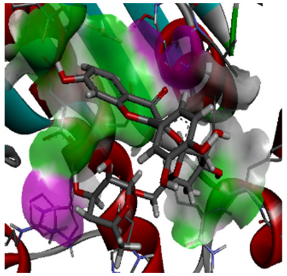	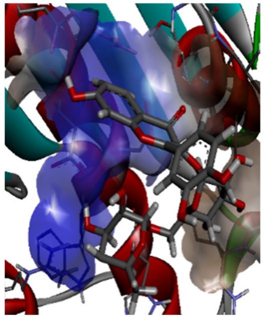	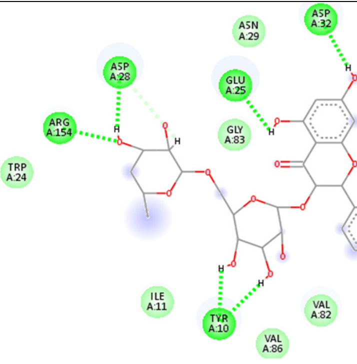

Penicillin	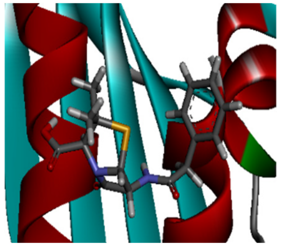	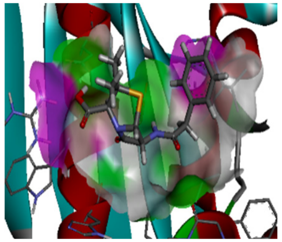	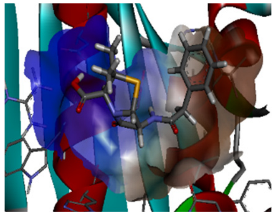	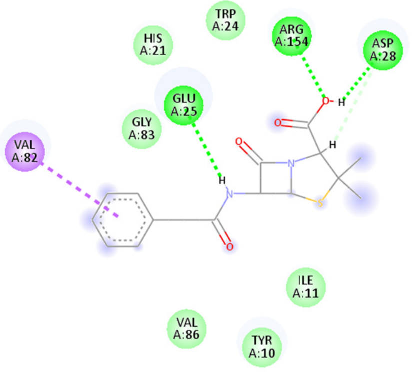

+: Van der Waals; +: Pi-Cation; +: Unfavorable Acceptor -Acceptor; +: Pi-Donor Hydrogen Bond; +: Pi-Sulfur; +: Pi-Sigma; +: Conventional Hydrogen Bond; +: Sulfur -X; +: Pi-Alkyl.
